# LINC01614 Promotes Oral Squamous Cell Carcinoma by Regulating FOXC1

**DOI:** 10.3390/genes15111461

**Published:** 2024-11-13

**Authors:** Hongze Che, Xun Zhang, Luo Cao, Wenjun Huang, Qing Lu

**Affiliations:** 1 School of Dentistry, Beihua University, Jilin 132013, China; 2 Department of Pathogenobiology, The Key Laboratory of Zoonosis, Chinese Ministry of Education, College of Basic Medicine, Jilin University, Changchun 130021, China; 3 VIP Integrated Department, Stomatological Hospital, Jilin University, Changchun 130021, China

**Keywords:** LINC01614, FOXC1, miR-138-5p, OSCC

## Abstract

**Background:** Long non-coding RNAs (lncRNAs) are pivotal mediators during the development of carcinomas; however, it remains to be investigated whether lncRNAs are implicated in oral squamous cell carcinoma (OSCC). **Methods:** In this study, quantitative real-time PCR was conducted for detecting the expression of LINC01614 in OSCC cell lines. The biological functions of LINC01614 were assessed by loss- and gain-of-function experiments conducted both in vivo and in vitro. Cellular proliferation, migration, and invasion were investigated herein, and dual luciferase reporter assays were additionally performed to explore the relationships among LINC01614, miR-138-5p, and Forkhead box C1 (FOXC1). **Results**: The research presented herein revealed that OSCC cells express high levels of LINC01614. Functional experiments employing cellular and animal models demonstrated that LINC01614 knockdown repressed the malignant phenotypes of OSCC cells, including their growth, invasiveness, and migration. Further investigation revealed that LINC01614 absorbs miR-138-5p miRNA by functioning as a competing endogenous RNA to downregulate the abundance of FOXC1. **Conclusions**: The findings revealed that LINC01614 contributes to the progression of OSCC by targeting the FOXC1 signaling pathway. The study provides insights into a novel mechanistic process to regulate the development of OSCC, and established a possible target for the therapeutic management of OSCC.

## 1. Introduction

Oral squamous cell carcinoma (OSCC) represents the leading form among head and neck cancers, having high morbidity and mortality rates [1,2], and a predisposition towards metastasis and recurrence. The occurrence of cancer involves highly complex processes and is primarily mediated by the interplay among environmental, genetic, and other factors [3,4,5]. Therefore, identification of the key altered genes that partake in the causation of OSCC is imperative in the development of new biomarkers or targeted therapies.

Long non-coding RNAs (lncRNAs) refer to ≥200-nt-long RNA molecules that lack protein-encoding potential [6,7]. Earlier reports have revealed that these transcripts exhibit aberrant abundance among carcinomas, and mediate tumor initiation and development by controlling the epigenetic machinery, regulating gene transcription, and sponging microRNAs [8,9]. Accumulating evidence indicates that aberrations in lncRNA expression and function can dysregulate gene expression, resulting in the onset, development, and metastasis of various tumors [10,11,12]. Some reports have confirmed that aberrations in lncRNA expression correlate with several human diseases, including cancer [13]. Research on the functions of lncRNAs in OSCC has increased gradually. For instance, it has been reported that AFAP1-AS1 aggravates the development of OSCC via ubiquitin-mediated proteolysis [14]. It has been additionally demonstrated that lncRNA CDKN2B-AS1 rs1333048 is associated with the development of late-stage tumors, but is not linked to disease occurrence in oral cancer [15]. LINC01614 is a 2180 nt-long lncRNA that is encoded by a gene present on chromosome 2 [16]. LINC01614 binds to GSK-3β to maintain β-catenin protein abundance, which markedly upregulates the Wnt/β-catenin signal transduction cascade among cancers of the pancreas [17]. It has been reported that LINC01614 induces FOXP1 expression via the inhibition of miR-217 to promote the progression of pulmonary adenocarcinoma [18]. However, the contribution of LINC01614 to the onset of OSCC and the intrinsic mechanism remain to be elucidated to date.

The current investigation determined the specific contributions of LINC01614 to the development of OSCC and elucidated the intrinsic mechanistic processes. The findings revealed that LINC01614 modulates FOXC1 expression in an miR-138-5p-dependent manner to enhance cellular viability, as well as their proliferative, migratory, and invasive potential, while inhibiting apoptosis through the competitive endogenous RNA (ceRNA) mechanism. The study provides insights for designing new biomarkers and possible druggable candidates for OSCC diagnostic management.

## 2. Materials and Methods

### 2.1. Cell Cultures and Transfections

Human oral keratinocyte (HOK) cells and OSCC cell lines CAL-27 and SCC-9 were procured from the Department of Pathogen Biology, School of Basic Medicine, Jilin University. Cell culture experiments were performed using DMEM (GIBCO, Waltham, MA, USA) comprising streptomycin/penicillin plus fetal bovine serum (FBS, 10%). Sangon Biotech (Shanghai, China) provided siRNAs against LINC01614 and FOXC1, the pcDNA-LINC01614 overexpression plasmid, inhibitors and mimics for miR-138-5p, and negative controls (NCs). Cellular transfection was conducted using Lipofectamine 2000 (Invitrogen, Waltham, MA, USA), as per protocols provided by the manufacturer.

### 2.2. Quantitative Real Time-Polymerase Chain Reaction (qRT-PCR)

TRIzol was adopted to isolate total RNA, which was subjected to cDNA synthesis using reverse transcription kits (TaKaRa Bio, San Jose, CA, USA). The qRT-PCR reaction systems were established using the Vazyme ChamQ Universal SYBR qPCR Master Mix. The 2^−ΔΔCt^ algorithm was adopted for quantification of relative target mRNA abundance. Sequences of the primers utilized in this experiment are enlisted in Table S1.

### 2.3. Cell Counting Kit-8 (CCK-8) Assays

Cell proliferative potential was appraised via CCK-8 kits (Klonda, Shanghai, China). Cell culture was performed for 48 h, following which the CCK-8 reagent (10 μL) was supplemented into the cells for a two-hour incubation period. The 450-nm optical density (OD) was finally detected utilizing a microplate reader.

### 2.4. Colony Formation Assays

Approximately 500 cells were cultured for 2 weeks inside 6-well culture plates, following which they were subjected to a 0.5-h fixation step with 4% paraformaldehyde (Beyotime, Shanghai, China). The cells were then stained with crystal violet (Beyotime, Shanghai, China), and the newly formed colonies were enumerated using an optical microscope (Nikon, Japan).

### 2.5. Transwell Assays

For the Transwell assays, a 37-ng/μL solution of Matrigel (500 μL, BD Biosciences, San Jose, CA, USA) was applied to the polycarbonate membranes, following which 5 × 10^4^ OSCC cells were inoculated into the system’s upper chamber and cultured with an FBS-free medium. The lower chamber was supplemented with FBS-supplemented medium. Cells present on the lower side of the membrane after incubation were subsequently counted to reflect their invasive ability. The fixation, staining, and observation steps were the same as those described in the “Colony formation assays” section.

### 2.6. Wound Healing Assays

After inoculating CAL-27 cells into 6-well plates, they were allowed to reach 80–90% fusion. Afterwards, the surface of the culture was scratched using a 200-μL pipette tip, following which they were rinsed with PBS and transferred to a medium without FBS. At 0 and 24 h post-scratch, the healing status of the wound was appraised.

### 2.7. Flow Cytometry

For the flow cytometric analysis, 5 × 10^4^ CAL-27 and SCC-9 cells were separately collected from each group and digested. The cells were subjected to double staining with the fluorescein isothiocyanate (FITC)-Annexin V/propyl iodide (PI) double dye, as per the protocols provided by the manufacturer. The population of apoptotic cells in the experimental cohort was assessed in relation to that of the NC cohort by flow cytometric analyses.

### 2.8. Dual-Luciferase Reporter Gene Assays

After inoculation into 96-well plates, CAL-27 cells were co-transfected with miR-138-5p mimics or NCs and plasmids expressing the luciferase gene. The plasmids harbored genes encoding WT or mutant LINC01614 (LINC01614-WT or LINC01614-MUT), and the 3ʹ-untranslated region of the WT or mutant FOXC1 (FOXC1-WT or FOXC1-MUT). A Luciferase Reporter Detection System (Promega, Madison, WI, USA) was utilized to determine luciferase activity two days after transfection as per protocols provided by the manufacturer.

### 2.9. Subcellular Fractionation Assays

The FastPure cytoplasmic and nuclear RNA purification kit (ECOTOP Scientific Co., Ltd., Guangzhou, China) was employed for extraction of RNA that resided in cytoplasm and nuclei. The processes of total RNA extraction, reverse transcription, and qRT-PCR followed the methods described above.

### 2.10. In Vivo Experiments

Vital River (Beijing, China) provided six-week-old nude mice, which were raised within an experimental setting devoid of specific pathogens. The mice were subcutaneously injected with 0.1 mL PBS containing 1 × 10^6^ re-suspended CAL-27 cells expressing si-LINC01614 or LINC01614. The tumor volumes were calculated at 7-day intervals with the equation: tumor volume = [(width) × (height)^2^]/2. At 28 days post-injection, the animals were sacrificed, the tumor weights were measured, and additional experiments were conducted.

### 2.11. Statistical Analyses

The mean ± standard deviation (SD) was calculated for all statistical data and analyzed with GraphPad Prism, version 6. All the experiments in this study were repeated at least thrice. Student’s t-tests (unpaired, two-tailed) were implemented to compare the differences between two groups, with *p* < 0.05 signifying statistical significance.

## 3. Results

### 3.1. OSCC Cells Exhibited Aberrantly High LINC01614 Expression

**Figure 1:**
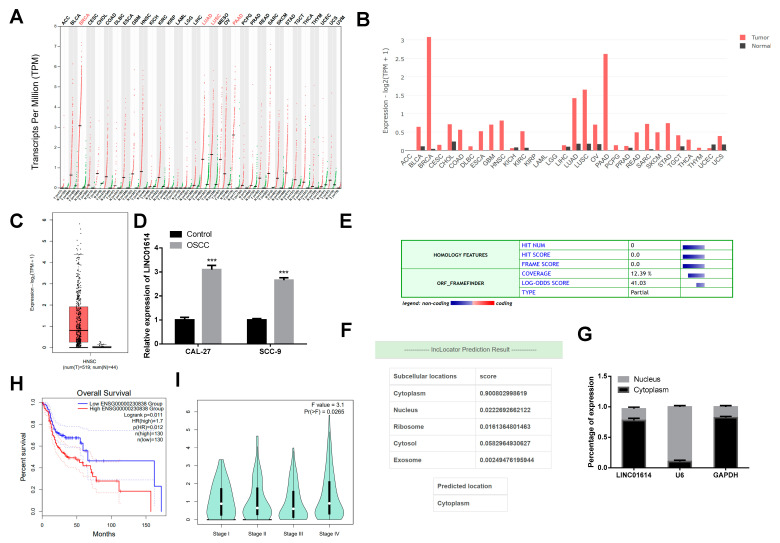
LINC01614 is upregulated in OSCC. (**A**,**B**) Expression patterns of LINC01614 in normal and cancer tissues, as reported in the GEPIA database. (**C**) Expression patterns of LINC01614 in HNSC based on data retrieved from the GEPIA database. (**D**) Expression levels of LINC01614 in CAL-27 and SCC-9 OSCC cell lines and control HOK cells. (**E**) The protein-coding potential of LINC01614 was assessed using the CPC tool. (**F**) Subcellular localization of LINC01614, as determined using the lncLocator. (**G**) Determination of the cytoplasmic and nuclear expression of LINC01614 in CAL-27 cells by qRT-PCR. (**H**) Relationship between the expression of LINC01614 and overall survival, as determined by Kaplan–Meier analysis. (**I**) Relationship between the stage of OSCC tumors and LINC01614 expression. *** *p* < 0.001.

LINC01614 abundance was analyzed based on relevant data of the GEPIA database. According to the analysis, LINC01614 is typically overexpressed in most cancers (Figure 1A,B), including head and neck squamous cell carcinoma (HNSC) (Figure 1C). The expression levels of LINC01614 in CAL-27 and SCC-9 cell lines were additionally assessed by qRT-PCR, which revealed that LINC01614 was upregulated in OSCC cells, relative to the control (Figure 1D). Analysis with the Coding Potential Calculator revealed that LINC01614 is devoid of protein-coding potential (Figure 1E). Further LINC01614 subcellular localization prediction with the lncLocator webtool revealed that it is primarily expressed in the cytoplasmic compartment, which implied that LINC01614 may function as a ceRNA (Figure 1F). Consistent with the prediction results, karyoplasmic separation further verified that LINC01614 was mostly expressed in the cytoplasmic compartment of OSCC cells (Figure 1G). Kaplan–Meier curves further elucidated that the LINC01614 upregulation correlated with dismal patient survival rates (Figure 1H), and that the expression of LINC01614 tended to decrease with the progression of OSCC (Figure 1I).

### 3.2. LINC01614 Promoted OSCC Cell Growth, Invasiveness, and Migratory Ability

**Figure 2:**
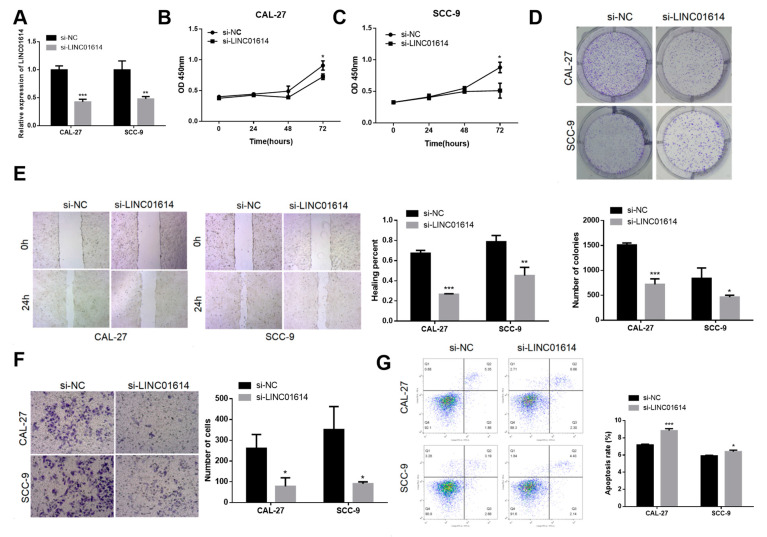
LINC01614 promoted the proliferation, migration, and invasion of OSCC cells in vitro. (**A**) Detection of the transfection efficacy of si-LINC01614 in OSCC cells. (**B**–**D**) Growth of OSCC cells with LINC01614 knockdown as determined by CCK-8 and colony formation assays. (**E**) Effects of si-LINC01614 on the migration of OSCC cells post-transfection, as determined by wound healing assays. (**F**) Alterations in the invasive potential of OSCC cells following the downregulation of LINC01614 expression, as determined by Transwell assays. (**G**) Effects of LINC01614 downregulation on the regulation of apoptosis in OSCC cells. * *p* < 0.05, ** *p* < 0.01, and *** *p* < 0.001.

LINC01614 knockdown efficacy in SCC-9 and CAL-27 cells was validated through qRT-PCR (Figure 2A). The cell viability analyses indicated that LINC01614 downregulation suppressed SCC-9 and CAL-27 cell growth (Figure 2B,C). As revealed by the colony formation assays, the downregulation of LINC01614 suppressed colony formation in the two cell lines (Figure 2D). The impact of LINC01614 silencing on OSCC cell migratory and invasive potential was further evaluated via wound healing and Transwell experiments. The findings revealed that LINC01614 downregulation significantly suppressed the migratory and invasive potential of CAL-27 and SCC-9 cell lines (Figure 2E,F). The flow cytometry experiments revealed an elevation in percentage of apoptotic cells following the downregulation of LINC01614, relative to that of the control group (Figure 2G). Altogether, the observations suggested that LINC01614 accelerated the growth, migration, and invasiveness of OSCC cell lines.

### 3.3. Si-LINC01614 Inhibited the Development of OSCC via Regulating miR-138-5p

**Figure 3:**
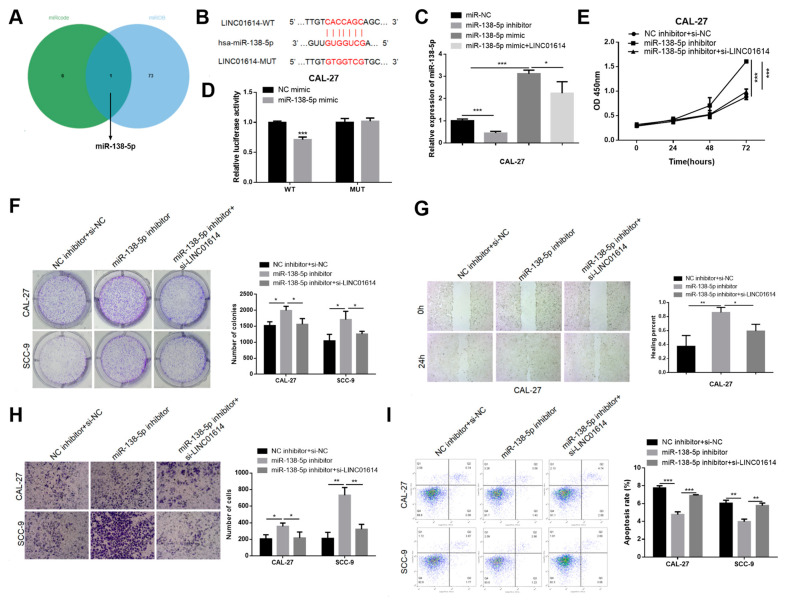
Si-LINC01614 suppressed the progression of OSCC by targeting miR-138-5p (**A**) The consensus results obtained from the databases, miRCode and miRDB, are depicted in the intersection area of the Venn diagram. (**B**) Prediction of the miR-138-5p binding site of LINC01614 using miRDB. (**C**) Alterations in the expression of miR-138-5p following transfection with miR-138-5p mimics or inhibitors, or LINC01614. (**D**) Effects of the miR-138-5p and NC mimics on the luciferase activities of the wild-type and mutant LINC01614 as determined by dual luciferase reporter gene analysis. (**E**,**F**) Results of CCK-8 and colony formation assays of OSCC cells following transfection with the miR-138-5p inhibitor, miR-138-5p inhibitor+si-LINC01614, or control. (**G**) Determination of the migration of OSCC cells transfected with the miR-138-5p inhibitor or miR-138-5p inhibitor+si-LINC01614 and control cells by wound healing assays. (**H**) Effects of the miR-138-5p inhibitor on the invasion of CAL-27 and SCC-9 cells with LINC01614 knockdown as determined by Transwell assays. (**I**) Apoptotic potential of OSCC cells following co-transfection with the miR-138-5p inhibitor and si-LINC01614 or NC. * *p* < 0.05, ** *p* < 0.01, and *** *p* < 0.001.

We subsequently determined whether LINC01614 functions as a ceRNA and sponges microRNAs (miRNAs). To this end, the possible targets of LINC01614 were predicted from different databases using bioinformatics webservers. The findings of target prediction analyses elucidated that miR-138-5p was the only candidate that could be targeted by LINC01614 (Figure 3A). The putative binding sites of miR-138-5p and LINC01614 are depicted in Figure 3B. The transfection efficacy of the miR-138-5p inhibitors and mimics in CAL-27 cells was additionally determined (Figure 3C). The findings indicated that the upregulation of LINC01614 downregulated miR-138-5p expression (Figure 3C).

Dual-luciferase reporter assays were subsequently conducted for verifying whether LINC01614 is capable of binding to miR-138-5p. The luciferase activity of cells transfected with the mutant LINC01614 (LINC01614-MUT), which mimicked miR-138-5p, was comparable to that of the control setup (Figure 3D). The results implied that LINC01614 regulates miR-138-5p function through direct binding in OSCC cells.

**Figure 4:**
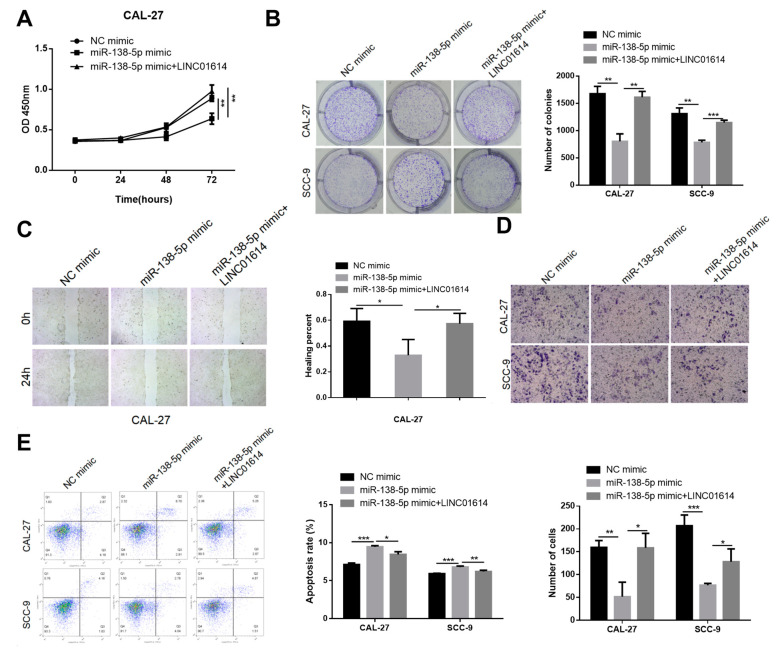
LINC01614 expression counteracted the effects of miR-138-5p upregulation on OSCC cells. (**A**,**B**) Alterations in the proliferative potential of OSCC cells following transfection with miR-138-5p mimics or co-transfection with miR-138-5p mimics and LINC01614, as determined by colony formation and CCK-8 assays. (**C**,**D**) Effects of LINC01614 on the migratory and invasive potential of OSCC cells following transfection with miR-138-5p mimics, as determined by wound healing and Transwell assays. (**E**) Detection of apoptosis in OSCC cells overexpressing LINC01614 or NC following transfection with miR-138-5p mimics or NC by flow cytometry. * *p* < 0.05, ** *p* < 0.01, and *** *p* < 0.001.

Salvage assays were subsequently employed for determining whether LINC01614 modulates the functions of miR-138-5p in OSCC cells. The inhibition of miR-138-5p significantly upregulated cellular proliferation, while LINC01614 knockdown counteracted this effect to a certain degree, as demonstrated by colony formation and cell viability assays (Figure 3E,F). LINC01614 downregulation markedly reduced the enhanced migration and invasiveness of both cell types via the antagonism of miR-138-5p, as indicated by the wound healing and Transwell assays (Figure 3G,H). Flow cytometric analyses elucidated that the anti-apoptotic potential of miR-138-5p in the CAL-27 cell line was restored post-co-transfection with LINC01614 (Figure 3I). Additionally, LINC01614 overexpression counteracted the repression of cellular growth, migration, and invasiveness by miR-138-5p mimics, and suppressed the induction of apoptosis (Figure 4A–E). Altogether, the findings implied that the carcinogenic effects of LINC01614 are mediated via the regulation miR-138-5p expression.

### 3.4. MiR-138-5p Targets FOXC1 to Downregulate FOXC1 Expression

**Figure 5:**
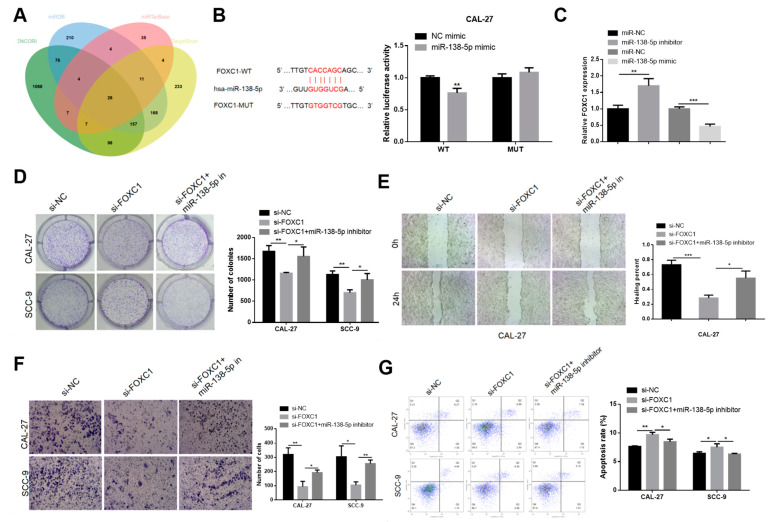
MiR-138-5p targets FOXC1 to negatively regulate the expression of FOXC1. (**A**) Prediction of the potential target genes of miR-138-5p using the ENCORI, TargetScan, miRDB, and miRTarBase databases. (**B**) The effects of co-transfection with miR-138-5p mimics/NC and FOXC1-WT or FOXC1-MUT were examined using dual-luciferase reporter gene assays. (**C**) Effects of FOXC1 expression in OSCC cells transfected with miR-138-5p mimics or inhibitors. (**D**–**G**) Effects of co-transfection with the miR-138-5p inhibitor and control or si-FOXC1, on the growth, migration, invasion, and apoptosis of OSCC cells, as determined by colony formation, wound healing, and Transwell assays, and flow cytometric analyses. * *p* < 0.05, ** *p* < 0.01, and *** *p* < 0.001.

The molecular components downstream of miR-138-5p were identified from four miRNA target prediction databases, including TargetScan. The 26 potential target genes consistently identified across the four databases have been depicted in a Venn diagram (Figure 5A). Further screening revealed that the FOXC1 gene could be a downstream target of miR-138-5p. The dual-luciferase reporter gene assays further elucidated that luciferase activity was repressed by the miR-138-5p mimics in the presence of the wild-type FOXC1 reporter gene, but remained unaltered in the presence of the mutant FOXC1 reporter gene (Figure 5B). Altogether, the findings revealed that miR-138-5p directly targets FOXC1, and is in turn sponged by LINC01614. In addition, the overexpression and downregulation of miR-138-5p induced the downregulation and overexpression of FOXC1, respectively (Figure 5C). The colony formation assays elucidated that FOXC1 knockdown suppressed the growth of the CAL-27 and SCC-9 cell lines, while the aberrant expression of miR-138-5p alleviated the repressive influence of FOXC1 knockdown on cell growth to a certain extent (Figure 5D). The miR-138-5p inhibitors also revived the reduction of the migratory and invasive potential of CAL-27 and SCC-9 cell lines and increased apoptosis following the downregulation of FOXC1 to a certain extent (Figure 5E–G).

### 3.5. FOXC1 Silencing Counteracted the Promoting Effects of LINC01614 Overexpression in OSCC Cells

**Figure 6:**
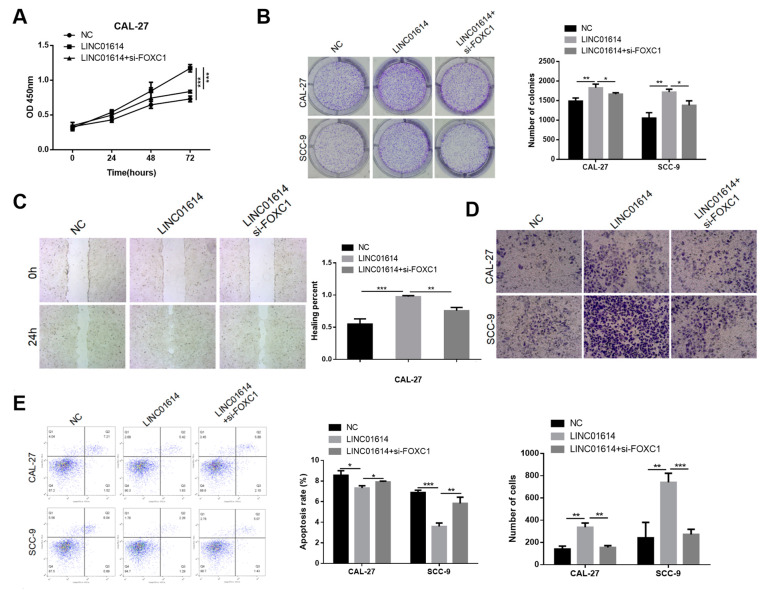
FOXC1 silencing counteracted the promoting effects of LINC01614 overexpression in OSCC cells. (**A**,**B**) Proliferative ability of OSCC cells (LINC01614, LINC01614+si-FOXC1, or NC) as determined by CCK-8 and colony formation assays. (**C**) Effects of co-transfection with LINC01614 and control or si-FOXC1 on the migratory potential of CAL-27 cells by wound healing assays. (**D**) Determination of the invasive potential of CAL-27 or SCC-9 cells transfected with LINC01614 or NC post-transfection with si-FOXC1 by Transwell assays. (**E**) Detection of the apoptosis of OSCC cells transfected with LINC01614 or LINC01614+si-FOXC1 and control OSCC cells by flow cytometry. * *p* < 0.05, ** *p* < 0.01, and *** *p* < 0.001.

We subsequently investigated whether LINC01614 can alter the expression of FOXC1 via its regulatory effect on miR-138-5p. The findings revealed that the upregulation of LINC01614 increased the proliferative potential (Figure 6A,B), migration, and invasiveness of SCC-9 and CAL-27 cell lines (Figure 6C,D), while inhibiting cellular apoptosis (Figure 6E); however, the effects of LINC01614 were partially counteracted by FOXC1 knockdown. These findings implied that LINC01614 accelerated the development of OSCC via the miR-138-5p/FOXC1 pathway.

### 3.6. LINC01614 Accelerated the Development of OSCC In Vivo

**Figure 7:**
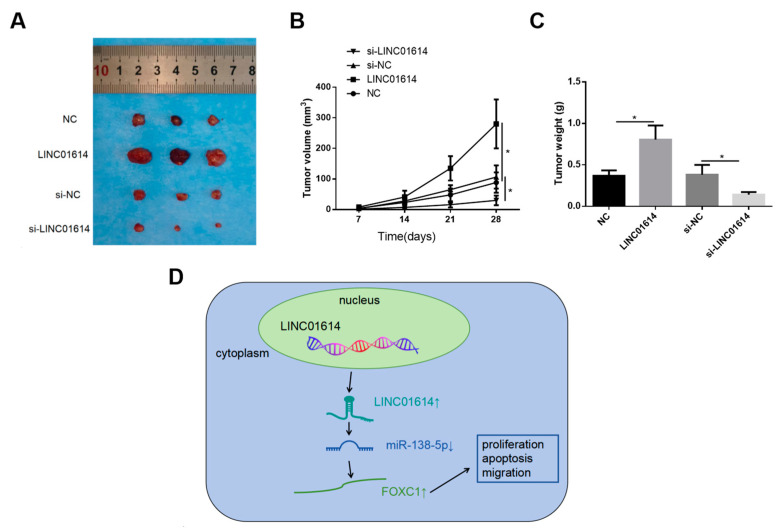
LINC01614 promoted the progression of OSCC in vivo. (**A**) Representative images of the subcutaneous OSCC tumors of nude mice. (**B**,**C**) Volumes and weights of the OSCC tumors. (**D**) Schematic depicting the role of the LINC01614/miR-138-5p/FOXC1 axis in the progression of OSCC. * *p* < 0.05.

CAL-27 cells were subcutaneously injected to construct the nude mouse model of transplanted tumors. The animals were sacrificed 4 weeks post-injection, following which the tumor tissues were collected. The findings revealed that LINC01614 downregulation significantly inhibited tumor development, relative to that of the control. Similarly, the tumor weights and volumes decreased significantly in the cohort that received si-LINC01614, relative to those of the control. Additionally, there was a significant acceleration in tumor growth following the upregulation of LINC01614, relative to that of the control (Figure 7A–C). Altogether, these findings demonstrated that LINC01614 can promote tumor growth in vivo.

## 4. Discussion

In recent years, mounting evidence has unveiled the functions of lncRNAs in regulating cellular function, differentiation, and the progression of various diseases, including cancer. They influence these processes via numerous mechanistic processes, namely, epigenetic control, chromatin remodeling, and regulation of the processes of transcription and post-transcription [19,20].

There are extensive reports on the critical regulatory roles of lncRNAs in cancer pathways [21,22]. By interacting with chromatin and proteins, lncRNAs can modulate RNA splicing and control transcriptional and post-transcriptional gene expression. Additionally, lncRNAs can modulate regulatory signaling cascades and mediate the epigenetic regulation of gene expression to regulate numerous cellular functions [23]. Among these, the ceRNA hypothesis posits that the interactions among lncRNAs, miRNAs, and mRNAs enable lncRNAs to modulate mRNAs via the competitive inhibition of miRNAs, facilitated by miRNA response elements (MREs) [24,25]. Several reports have confirmed the role of LINC01614 in cancer. For instance, a previous investigation reported that LINC01614 acts as a ceRNA and promotes the proliferative and invasive potential of OS cells through the miR-520a-3p/SNX3 pathway [26]. Additionally, SP1 can induce LINC01614 expression to enhance the malignant development of gliomas via the miR-383/ADAM12 pathway [27]. Nevertheless, the expression profiles and functions of LINC01614 in the development of OSCC remain poorly understood to date. The current investigation demonstrated that LINC01614 is upregulated in OSCC cells, which led us to speculate that LINC01614 might be associated with the pathogenesis of OSCC.

The cell function experiments performed in this study revealed that LINC01614 enhanced the growth, migration, and invasiveness of OSCC cell lines, while inhibiting apoptosis. The in vivo experiments revealed that LINC01614 accelerated the onset of OSCC tumors in vivo, indicating its role in accelerating the progression of OSCC.

The possible functions of LINC01614 in OSCC were further explored by initial determination of its intracellular distribution, which revealed that the cytoplasmic compartment is its primary location. It led us to speculate that LINC01614 might act as an miRNA sponge and absorb miRNAs. The results of bioinformatics analyses revealed the presence of complementary LINC01614 binding sites in miR-138-5p. The interaction of LINC01614 with miR-138-5p was subsequently detected by dual-luciferase reporter assays, which confirmed that miR-138-5p exerts a cancer-suppressive effect in various cancers. The miR-138-5p miRNA can suppress the vascular simulation of HepG2 and Hep3B cells via inhibition of the HIF-1α/VEGFA cascade in liver cancer [28]. MiR-138-5p also suppresses the growth and invasiveness of bladder cancer via the downregulation of BIRC5 expression [29]. Another study demonstrated that by targeting the NFIB-Snail1 pathway, miR-138-5p might critically regulate the migratory potential and resistance of colorectal cancer cells to chemotherapy [30]. The current investigation established that LINC01614 promoted the proliferative and metastatic potential of OSCC through its role as an miR-138-5p sponge.

We subsequently identified that FOXC1 is a downstream target of miR-138-5p, and that FOXC1 mRNA directly interacts with miR-138-5p in OSCC cells. The findings suggested the possible modulation of FOXC1 expression by LINC01614, which competes with miR-138-5p for FOXC1 mRNA. It has been recently identified that FOXC1, which is encoded by a gene located on chromosome 6p25, regulates several human cancers [31,32]. FOXC1 can induce phenotypic plasticity by binding to enhancer elements and accelerating the transition of bladder cancer cells to the cisplatin-resistant phenotype via a mutation-independent manner [33]. FOXC1 promotes the development of cancer stem-like characteristics in non-small cell lung cancer by upregulating ꞵ-catenin expression [34]. The series of remedial experiments conducted herein revealed that the regulatory roles of LINC01614 were counteracted by upregulating miR-138-5p or downregulating FOXC1 expression. This led us to speculate that LINC01614 can promote the progression of OSCC via the induction of FOXC1 expression by sponging miR-138-5p (Figure 7D). The observations obtained herein provide detailed perspectives and enhance our understanding of the progression of OSCC.

Altogether, the present study revealed that LINC01614 promotes OSCC by enhancing the growth, migration, and invasiveness of OSCC cell lines. We additionally identified that LINC01614 functions as a ceRNA to upregulate FOXC1 in OSCC cells by sponging miR-138-5p. The results obtained herein offer detailed insights into novel and potential targets for the therapeutic management of OSCC.

## Data Availability

The original contributions presented in the study are included in the article, further inquiries can be directed to the corresponding author.

## Supplementary Materials

The following supporting information can be downloaded at: https://www.mdpi.com/article/10.3390/genes15111461/s1, Table S1: Primer sequences used for qRT-PCR.
