# Supplementary material for: LINC01614 Promotes Oral Squamous Cell Carcinoma by Regulating FOXC1
Source: Genes (Basel). 2024 Nov 13;15(11):1461. doi: 10.3390/genes15111461 (PMC11593781; doi:10.3390/genes15111461)
Supplement: Supplementary file 1 [file genes-15-01461-s001.zip › genes-3292879-supplementary materials.pdf]

Table S1. Primer sequences used for qRT-PCR.

Gene	Sequence (5′-3′)
LINC01614 FORWARD	CACGGAGAATCTAAGACACCAGGAG
LINC01614 REVERSE	CTTGGCTTCGCTCTTGGTTGAC
FOXC1 FORWARD	TTGCCTTCTTCCTTGCCTCTCAC
FOXC1 REVERSE	GCGACTTTCATAAACGGGGACTTTC
GAPDH FORWARD	GAACGGGAAGCTCACTGG
GAPDH REVERSE	GCCTGCTTCACCACCTTCT
U6 FORWARD	GCTTCGGCAGCACATATACTAAAAT
U6 REVERSE	CGCTTCACGAATTTGCGTGTCAT
miR-138-5p FORWARD	AACACGTGAGCTGGTGTTGTGA
miR-138-5p REVERSE	ATCCAGTGCAGGGTCCGAGG
